# Extracellular Vesicles from Caveolin-Enriched Microdomains Regulate Hyaluronan-Mediated Sustained Vascular Integrity

**DOI:** 10.1155/2015/481493

**Published:** 2015-09-10

**Authors:** Tamara Mirzapoiazova, Frances E. Lennon, Bolot Mambetsariev, Michael Allen, Jacob Riehm, Valeriy A. Poroyko, Patrick A. Singleton

**Affiliations:** ^1^Department of Medicine, Section of Pulmonary and Critical Care, Pritzker School of Medicine, The University of Chicago, Chicago, IL, USA; ^2^Department of Medicine, Section of Hematology/Oncology, Pritzker School of Medicine, The University of Chicago, Chicago, IL, USA; ^3^Department of Surgery, Pritzker School of Medicine, The University of Chicago, Chicago, IL, USA; ^4^Department of Anesthesia and Critical Care, Pritzker School of Medicine, The University of Chicago, Chicago, IL, USA

## Abstract

Defects in vascular integrity are an initiating factor in several disease processes. We have previously reported that high molecular weight hyaluronan (HMW-HA), a major glycosaminoglycan in the body, promotes rapid signal transduction in human pulmonary microvascular endothelial cells (HPMVEC) leading to barrier enhancement. In contrast, low molecular weight hyaluronan (LMW-HA), produced in disease states by hyaluronidases and reactive oxygen species (ROS), induces HPMVEC barrier disruption. However, the mechanism(s) of sustained barrier regulation by HA are poorly defined. Our results indicate that long-term (6–24 hours) exposure of HMW-HA induced release of a novel type of extracellular vesicle from HLMVEC called enlargeosomes (characterized by AHNAK expression) while LMW-HA long-term exposure promoted release of exosomes (characterized by CD9, CD63, and CD81 expression). These effects were blocked by inhibiting caveolin-enriched microdomain (CEM) formation. Further, inhibiting enlargeosome release by annexin II siRNA attenuated the sustained barrier enhancing effects of HMW-HA. Finally, exposure of isolated enlargeosomes to HPMVEC monolayers generated barrier enhancement while exosomes led to barrier disruption. Taken together, these results suggest that differential release of extracellular vesicles from CEM modulate the sustained HPMVEC barrier regulation by HMW-HA and LMW-HA. HMW-HA-induced specialized enlargeosomes can be a potential therapeutic strategy for diseases involving impaired vascular integrity.

## 1. Introduction

Vascular integrity (i.e., the maintenance of blood vessel continuity) is required for normal cardiovascular homeostasis [1, 2]. Several mechanisms regulate basal vascular integrity including the endothelial glycocalyx and endothelial cell-cell junctions which are controlled by tight junctions, adherens junctions, and caveolin-enriched microdomains (CEM), a subset of lipid rafts containing the scaffolding protein caveolin-1 [1, 3–7]. Certain pathologies, including atherosclerosis, sepsis, ischemia/reperfusion, acute lung injury, diabetes, and cancer metastasis, induce degradation of the glycocalyx and disruption of EC-EC junctions causing leakage of fluids and proteins into the underlying tissue [1, 2, 4, 8, 9]. Therefore, understanding the mechanism(s) of EC barrier regulation can have important clinical utility.

The major nonsulfated glycosaminoglycan in most tissues, hyaluronan (HA), plays a fundamental role in the maintenance of vascular integrity [4, 8, 10–17]. We have previously demonstrated that HA and its major cell surface receptor, CD44, regulate pulmonary vascular integrity and that HMW-HA could potentially be utilized as a therapeutic intervention for defects in vascular integrity [5, 18, 19]. Specifically, HMW-HA (~1 million Da) binds to the transmembrane receptor, CD44s (standard form), in CEM which initiates a rapid signal transduction cascade. CD44s transactivates the barrier enhancing S1P1 receptor within CEM which results in the serine/threonine kinase, Akt-mediated activation of the Rac1 guanine nucleotide exchange factor, Tiam1, and Rac1-GTP formation leading to cortical actin formation and strengthening of EC-EC contacts. Further, HMW-HA recruits several other actin regulatory proteins to CEM including protein S100-A10, filamin-A, and filamin-B which enhance cortical actin formation and vascular integrity. In contrast to HMW-HA, LMW-HA (~2,500 Da) binds to and activates the HA receptor, CD44v10 (variant 10) in CEM. CD44v10 then transactivates the barrier disruptive S1P3 receptor. These events promote RhoA guanine nucleotide exchange factor (RhoGEF) activation and RhoA-GTP formation which stimulates the serine/threonine kinase, rho kinase (ROCK). This leads to actin stress fiber formation and EC barrier disruption. However, the long-term sustained pulmonary EC barrier regulatory mechanism(s) by HA is poorly defined.

Extracellular vesicles (EVs) come in several types including microvesicles, exosome-like vesicles, exosomes, and membrane particles [20, 26–29]. Each type has specific protein markers and is formed from either budding of the plasma membrane or release of intracellular multivesicular endosomes [20]. EVs range in size from 20 to 1,000 nm and are released by a variety of cells including EC [20]. EVs are believed to be a means of cell-cell communication and can transport proteins, mRNA, and miRNA to target cells [20, 30]. Enlargeosomes are specialized vesicles enriched in AHNAK and annexin II that have been observed intracellularly, fusing to the plasma membrane and shedding from the plasma membrane [21–23]. However, the role(s) of EV in HA-mediated sustained vascular integrity are unknown.

In the current study, we investigated the mechanism of HA-mediated long-term EC barrier regulation. We have identified that ~6 hours after HA addition, human EC differentially release EVs that contain caveolin-1 and are regulated by CEM. Utilizing several novel techniques including atomic force microscopy and nanosight nanoparticle tracking analysis (NTA), we have characterized these EVs as exosomes (for LMW-HA) and enlargeosomes (for HMW-HA). Importantly, isolated LMW-HA-induced exosomes promote EC barrier disruption while isolated HMW-HA-induced enlargeosomes induce EC barrier enhancement. Inhibiting HMW-HA-induced enlargeosome release reduces the sustained barrier enhancing properties of HMW-HA. These EVs differentially express HA, HA binding proteins, and microRNA which can potentially contribute to their differential EC barrier regulatory effects. Our findings demonstrate an important mechanism of HA-induced sustained vascular integrity through differential release of EV from human EC. Further, utilization of isolated HMW-HA-induced specialized enlargeosomes and/or their bioactive components can be a potential therapeutic strategy for diseases involving impaired vascular integrity.

## 2. Materials and Methods

### 2.1. Antibodies and Reagent

Antibodies utilized in this study include anti-CD9 (Santa Cruz Biotechnology Inc., Dallas, TX), anti-CD63 (Abcam, Cambridge, MA), anti-CD81 (GeneTex Inc., Irvine, CA), anti-AHNAK 1 (Thermo Scientific, Waltham, MA), anti-HABP2 (Abnova, Walnut, CA), anti-CD44 (IM7 clone) (BD Biosciences), anti-CD44v10 (Novus Biologicals), and anti-actin (Sigma). Hyaluronic acid (sodium salt from* Streptococcus zooepidemicus*), LPS, ATP, and ionomycin were purchased from Sigma. Reagents for SDS-PAGE electrophoresis were purchased from Bio-Rad (Richmond, CA) and Immobilon-P transfer membrane was purchased from Millipore (Millipore Corp., Bedford, MA).

### 2.2. Human Pulmonary Microvascular Endothelial Cell (HPMVEC) Culture

Primary human pulmonary microvascular cells were purchased from Lonza and maintained in EBM-2 growth media supplemented with EGM-2-MV Bulletkit and 10% fetal bovine serum. Cells were maintained at 37°C in a humidified atmosphere of 5% CO_2_-95% air and used for experimentation at passages 3–6.

### 2.3. Preparation of LMW-HA

LMW-HA was prepared similar to that as we have previously described [9, 31]. Briefly 500 mg of hyaluronan sodium salt from* Streptococcus zooepidemicus* was digested with 20,000 units of bovine testicular hyaluronidase (Type VI-S, lyophilized powder, 3,000–15,000 units/mg (Sigma, H3631) in digestion buffer (0.1 M sodium acetate, pH 5.4, 0.15 M NaCl) for 24 h), and the reaction stopped with 10% trichloroacetic acid. The resulting solution was centrifuged in an Ultrafree-MC Millipore 5 kDa MW cutoff filter and the flow-through was dialyzed against distilled water for 24 h at 4°C in 500 Da cutoff Spectra-Por tubing (Pierce-Warriner, Chester, UK). LMW-HA was quantitated using an ELISA-like competitive binding assay with a known amount of fixed HA and biotinylated HA binding peptide (HABP) as the indicator (Echelon Inc). LMW-HA solutions were filtrated through 0.22 *μ*m filters and kept in sterile tubes. In some cases, both low and high MW HA were subject to boiling, proteinase K (50 *μ*g/mL) digestion, hyaluronidase SD digestion (*Streptococcus dysgalactiae*, NorthStar Bioproducts Associates of Cape Cod Inc., East Falmouth, MA (100741-1A), 100 mU/mL utilized), or addition of boiled (inactivated) hyaluronidase SD to test for possible protein/lipid contaminants. To test for endotoxin contamination of HA, a lipopolysaccharides (LPS) bioAssay ELISA kit (USBiological Life Sciences) was utilized. LMW-HA with HA standards (Sigma and Enzo Life Sciences) were run on 4–20% Tris/Borate/EDTA (TBE) gels and stained with Stains-All (Sigma) or Alcian blue to confirm LMW-HA purity and size.

### 2.4. Inhibition of Protein Expression in Human EC Utilizing siRNA

Human lung microvascular EC were transfected with siRNA against human annexin II mRNA (Santa Cruz Biotechnology, Santa Cruz, CA) using siPORT Amine as the transfection reagent (Ambion, TX) according to the protocol provided by Ambion. Cells (~40% confluent) were serum-starved for 1 hour followed by incubation with 250 nM of target siRNA (or scramble siRNA or no siRNA) for 6 hours in serum-free media. The serum-containing media were then added (10% serum final concentration) for 42 h before biochemical experiments and/or functional assays were conducted. Effective silencing of target protein expression was determined with immunoblot analysis of siRNA-transfected EC lysates using specific antibodies.

### 2.5. Extracellular Vesicle (EV) Isolation

EVs were isolated from cell-free media from treated EC for 0, 1, 2, 3, 6, 12, or 24 hours. The conditioned media were centrifuged at 300 ×g for 15 min, 2,000 ×g for 30 min, and 12,000 ×g for 45 min to remove whole cells and debris. The resultant supernatant was passed through a 0.22 mm filter sterilized Steritop (Millipore, Billerica, MA, USA) and then centrifuged at 100,000 ×g for 75 min (Thermo Fisher Scientific Ins., Asheville, NC, USA, Sorvall, SureSpin 630/36, fixed angle rotor). The pellet was resuspended in PBS, pH = 7.4, washed, and recentrifuged (100,000 ×g, 75 min). The pellet was then resuspended in PBS and overlayed on a discontinuous OptiPrep gradient (40, 20, 10, and 5% OptiPrep solution in 0.25 M sucrose, 10 mM Tris, pH 7.5) and centrifuged at 100,000 ×g for 16 h. The EVs float at a density of 1.10–1.12 g/mL OptiPrep. Fractions were collected from the top of the gradient, diluted with 10 mM Tris buffer, and centrifuged at 100,000 ×g for 3 h; the subsequent pellets were resuspended in PBS, pH = 7.4, and subjected to EV characterization [32, 33].

### 2.6. Immunoblotting

Extracellular vesicles from treated or untreated HPMVEC were incubated with IP buffer (50 mM HEPES (pH 7.5), 150 mM NaCl, 20 mM MgCl_2_, 1% Nonidet P-40 (NP-40), 0.4 mM Na_3_VO_4_, 40 mM NaF, 50 *μ*M okadaic acid, 0.2 mM phenylmethylsulfonyl fluoride, and 1 : 250 dilution of Calbiochem protease inhibitor mixture 3). The samples were then run on SDS-PAGE in 4–15% polyacrylamide gels, transferred onto Immobilon membranes, and developed with specific primary and secondary antibodies. Visualization of immunoreactive bands was achieved using enhanced chemiluminescence (Amersham Biosciences). In some instances, computer-assisted densitometry was utilized to quantitate immunoreactive bands.

### 2.7. Isolation of Exosomal RNA and RNA Analysis

Exosomal pellets were dissolved in lysis buffer from RNAs isolation kit mirVana (Ambion/Life Technologies, Carlsbad, CA) and processed for small RNA isolation using column based methods according to the manufacture's instruction. For detection and analysis of the extracted exosomal small RNAs, we used an Agilent 2100 Bioanalyzer with a small RNA chip kit (Agilent Technologies).

### 2.8. Transendothelial Electrical Resistance (TER)

For EC barrier function, human pulmonary microvascular endothelial cells were grown to confluence in polycarbonate wells containing evaporated gold microelectrodes (surface area, 10–3 cm^2^) in series with a large gold counter electrode (1 cm^2^) connected to a phase-sensitive lock-in amplifier as described previously. Measurements of transendothelial electrical resistance (TER) were performed using an electrical cell-substrate impedance sensing system (ECIS) (Applied BioPhysics Inc.). Briefly, current was applied across the electrodes by a 4,000-Hz AC voltage source with amplitude of 1 V in series with a 1 M*Ω* resistance to approximate a constant current source (1 *μ*A). As cells adhere and spread out on the microelectrode, TER increases (maximal at confluence), whereas cell retraction, rounding, or loss of adhesion is reflected by a decrease in TER. These measurements provide a highly sensitive biophysical assay that indicates the state of cell shape and focal adhesion. Values from each microelectrode were pooled at discrete time points and plotted versus time as the mean ± SE of the mean [34–37].

### 2.9. Atomic Force Microscopy (AFM)

AFM was utilized similar to that as we have previously described [34]. Briefly, AFM offers a novel way to analyze the morphology and size of EV [38]. Isolate EVs were diluted in PBS, pH = 7.4, and plated on mica. AFM (tapping mode) was then utilized on a DI multimode AFM (Digital Instruments, Santa Barbara, CA) using a V-shaped oxide-sharpened silicon nitride cantilever with an optical scanning speed of ~1.0 Hz to obtain surface topology maps.

### 2.10. Nanosight Nanoparticle Tracking Analysis (NTA)

EVs as described above were subjected to NTA analysis as previously described [39]. NTA utilizes the properties of both light scattering and Brownian motion in order to predict particle size distributions of dilute samples in liquid suspension. A 635 nm laser beam was passed through a prism-edged glass flat under the ultrathin sample chamber. The angle of incidence of the laser and the refractive index of the glass are designed to illuminate the sample chamber, and the light is refracted by particles in the sample solution. The scattered beam light from a sample of EV diluted in PBS, pH = 7.4, was visualized using a 20x magnification microscope objective fitted to a conventional optical microscope. A high-sensitivity complementary metal oxide semiconductor camera recorded 30 frames per second video of the scattered light. A triplicate of 60 s video files was recorded for each sample, advancing the sample solution to a new 100 *μ*m × 80 *μ*m × 10 *μ*m field of view for each recording. The Nanosight software (v3.0) was then used to identify and track individual particles on a frame-by-frame basis. The mean squared two-dimensional displacement of each tracked particle was recorded and, coupled with the temperature, viscosity of the suspension media, and the Stokes-Einstein equation, was used to estimate a sphere equivalent hydrodynamic radius. The software tracked multiple EVs, with parameters set by the user, to provide a particle size distribution and concentration of the sample.

### 2.11. Statistical Analysis

Results are expressed as mean ± standard deviation of three independent experiments. For data analysis, experimental samples were compared to controls by unpaired Student's *t*-test. For multiple-group comparisons, a one-way variance analysis (ANOVA) and post hoc multiple comparisons tests were used. Differences between groups were considered statistically significant when *P* value was less than 0.05. All statistical analyses were performed using the GraphPad Prism program (GraphPad Software Inc., USA).

## 3. Results

**Figure 1:**
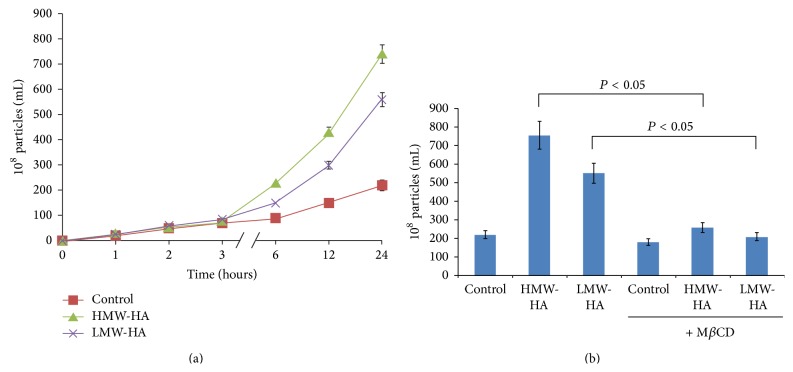
Determination of extracellular vesicle (EV) release kinetics and caveolin-enriched microdomain (CEM) dependence in human endothelial cells (EC). Panel (a): graphical representations of the kinetics of HA-induced EV release from human pulmonary microvascular endothelial cells (HPMVEC). Human EC monolayers were placed in serum-free media and the resulting media containing EV were collected at 0, 1, 2, 3, 6, 12, and 24 hours. The concentrations of EV were determined utilizing nanosight nanoparticle tracking analysis (NTA). HPMVEC exhibit basal secretion of EV (control). However, addition of 100 nM HMW-HA or 100 nM LMW-HA dramatically increased the production of EV starting at ~6 hours posttreatment. *N* = 3 per group and error bars = standard deviation. Panel (b): graphical representation of the role of CEM in HA-mediated EV production from human EC. HPMVEC monolayers were either untreated or treated with 5 mM methyl-*β*-cyclodextrin (M*β*CD, a CEM disrupting agent) with or without addition of 100 nM LMW-HA or 100 nM HMW-HA (24 hours). EVs were analyzed as described in Panel (a). Inhibiting CEM formation significantly inhibited both LMW-HA and HMW-HA-mediated EV secretion with *n* = 3 per group and error bars = standard deviation.

In order to identify potential mechanism(s) of hyaluronan- (HA-) mediated sustained human endothelial cell (EC) barrier function, we first determined the kinetics of HA-induced extracellular vesicle (EV) release. Human pulmonary microvascular EC (HPMVEC) were grown to confluence and placed in serum-free media, and no HA (control), 100 nm HMW-HA, or 100 nm LMW-HA were added for 0, 1, 2, 3, 6, 12, or 24 hours. Treated media were then collected and analyzed using nanosight nanoparticle tracking analysis (NTA) to determine EV concentrations (see Section 2). The results of Figure 1(a) indicate that HPMVEC secrete basal levels of EV. However, there is a dramatic increase in EV release from HPMVEC with either LMW-HA or HMW-HA addition starting at ~6 hours.

Since caveolin-1 has been implicated in EV release [40] and we have previously demonstrated that caveolin-enriched microdomains (CEM) are crucial for EC barrier function [5–7, 41, 42], we next examined whether CEM regulate HA-induced EV secretion in HPMVEC.  Figure 1(b) indicates that inhibiting CEM formation with methyl-beta-cyclodextrin (M*β*CD) inhibits both LMW-HA and HMW-HA-induced EV from HLMVEC (24-hour treatment) indicating the important role of this specialized plasma membrane microdomain in HA-induced EV secretion.

**Figure 2:**
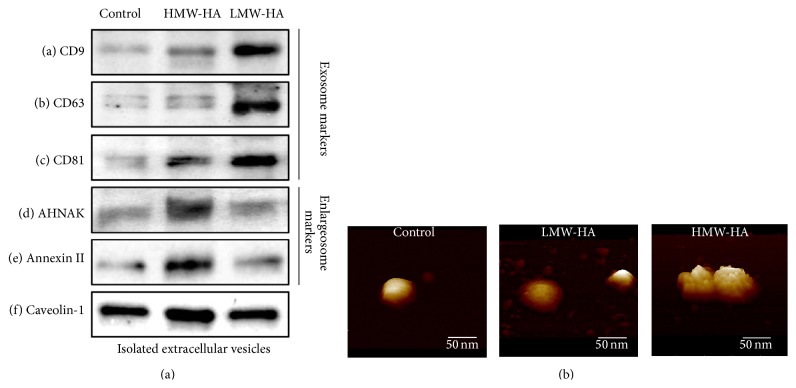
Identification of LMW-HA and HMW-HA-induced EV from human EC. Panel (a): biomarker analysis of HA-induced EV. HPMVEC were grown to confluence and switched to serum-free media and either no HA (control), 100 nM HMW-HA, or 100 nM LMW-HA for 24 hours. EVs were then isolated, run on SDS-PAGE, and immunoblotted with anti-CD9 (a), anti-CD63 (b), anti-CD81 (c), anti-AHNAK (d), antiannexin II (e), or anti-caveolin-1 (f) antibodies. LMW-HA-induced EV expressed exosome markers while HMW-HA-induced EV expressed enlargeosome markers. EV also expressed caveolin-1, a crucial component of caveolin-enriched microdomains (CEM). Panel (b): topographical images of HA-induced EV using atomic force microscopy (AFM). Isolated HA-induced EVs as described in Panel (a) were plated on mica and subjected to AFM analysis (see Section 2). The vesicles were never dried and are shown as imaged under buffer. Control EV and exosomes were round in shape and had a relatively smooth surface. In contrast, enlargeosomes were relatively round in shape but had a rough uneven surface topology. Control EV and exosomes had a diameter of ~50 nm which is consistent with the accepted exosome size range of 30–100 nm [20]. HMW-HA-induced enlargeosomes appeared slightly larger in comparison to exosomes.

We next characterized HA-induced EV using biomarker analysis and atomic force microscopy (AFM). The results of Figure 2(a) indicate that LMW-HA induces EV with biomarkers consistent with exosomes (CD9, CD63, and CD81). In contrast, HMW-HA induces EV with biomarkers consistent with a novel vesicle called an enlargeosome. Enlargeosomes are specialized vesicles enriched in AHNAK and annexin II that have been observed intracellularly, fusing to the plasma membrane and shedding from the plasma membrane [21–23]. Intrigued with these results, we further analyzed the HA-induced EV with AFM.  Figure 2(b) indicates control EV and exosomes are round in shape and have a relatively smooth surface. In contrast, enlargeosomes are relatively round in shape but have a rough uneven surface topology. Control EV and exosomes have a diameter of ~50 nm which is consistent with the accepted exosome size range of 30–100 nm [20]. HMW-HA-induced enlargeosomes appear slightly larger in comparison to exosomes.

**Figure 3:**
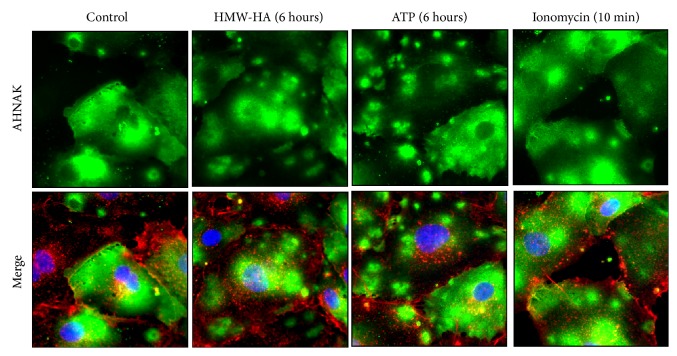
Analysis of HMW-HA-induced AHNAK redistribution in human EC. HPMVEC were grown to confluence on glass coverslips and treated with 100 nM HMW-HA for 6 hours. 50 *μ*M ATP (six hours) and 3 *μ*M ionomycin (10 minutes) were used as controls due to their previously reported ability to stimulate enlargeosome exocytosis [21–24]. The samples were then fixed in 4% paraformaldehyde and subjected to immunocytochemical analysis using AHNAK antibodies (green). Plasma membranes were labeled with Alexa Fluor 594 wheat germ agglutinin (red) and nuclei were stained with Hoechst 33342 (blue). Control HPMVEC exhibited diffuse cytoplasmic AHNAK staining. When stimulated with HMW-HA, intracellular AHNAK redistributed to a “vesicular” pattern which is similar to treatment with ATP and ionomycin.

To further investigate HMW-HA-induced enlargeosome dynamics in HLMVEC, confluent monolayers were treated with HMW-HA for 6 hours. ATP and ionomycin were used as controls due to their previously reported ability to stimulate enlargeosome exocytosis [21–24]. The samples were then fixed and subjected to immunocytochemical analysis using AHNAK antibodies (green). Plasma membranes were labeled with Alexa Fluor 594 wheat germ agglutinin (red) and nuclei were stained with Hoechst 33342 (blue). The results of Figure 3 indicate that control HPMVEC exhibit diffuse cytoplasmic AHNAK staining. When stimulated with HMW-HA, intracellular AHNAK redistributes to a “vesicular” pattern which is similar to treatment with ATP and ionomycin.

**Figure 4:**
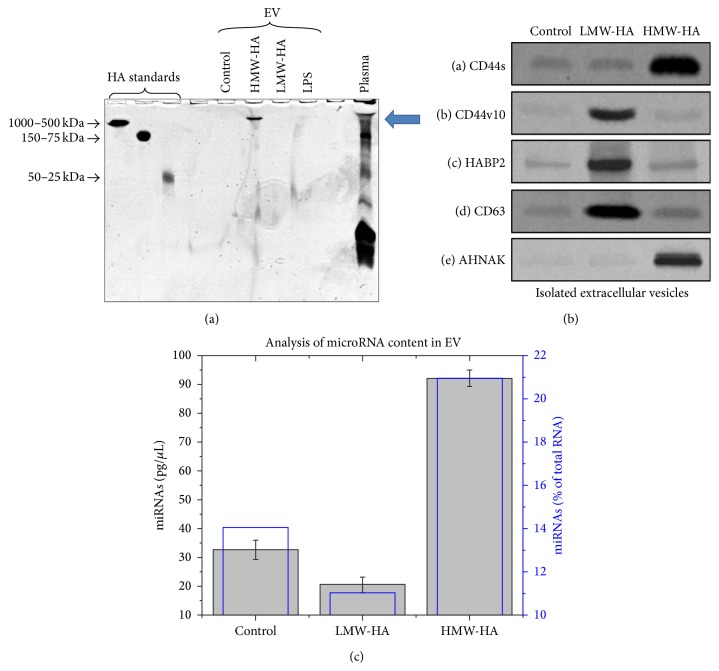
Characterization of potential bioactive components in HA-induced EV. Panel (a): HPMVEC were grown to confluence and switched to serum-free media and either no HA (control), 100 nM HMW-HA, 100 nM LMW-HA, or 500 ng/mL LPS for 24 hours. EVs were then isolated, run on 4–20% TBE gels, and stained with Alcian blue. Purified enlargeosomes contained HA of ~1 million Da (see arrow) consistent with HMW-HA which we have previously demonstrated to be barrier enhancing [4, 5, 7, 8]. In contrast, control, LMW-HA, or LPS-induced EV contained negligible HA. Human plasma was used as a control. Panel (b): HPMVEC were grown to confluence and switched to serum-free media and either no HA (control), 100 nM HMW-HA, or 100 nM LMW-HA for 24 hours. EVs were then isolated, run on SDS-PAGE, and immunoblotted with anti-CD44 (IM-7) (a), anti-CD44v10 (b), anti-HABP2 (c), anti-CD63 (d), or anti-AHNAK (e) antibodies. HMW-HA-induced enlargeosomes expressed the EC barrier enhancing CD44 isoform, CD44s (standard form) [4, 7]. In contrast, LMW-HA-induced exosomes expressed the EC barrier disrupting HA binding proteins, CD44 isoform CD44v10, and the extracellular serine protease, HABP2 [7, 25]. Basally secreted EV (control) had low expression of these molecules. Panel (c): isolated EVs as described in Panel (b) were subjected to RNA isolation and analysis (see Section 2). Compared to control EV, LMW-HA-induced EV had less total RNA and microRNA while HMW-HA-induced enlargeosomes had ~2-fold higher levels of total RNA and microRNA.

Considering the results of Figures 2 and 3, we further investigated these HA-induced EVs to determine the presence or absence of potential bioactive agents. Interestingly, Alcian blue stained TBE gels revealed that purified enlargeosomes contain HA of ~1 million Da (see arrow) consistent with HMW-HA which we have previously demonstrated to be barrier enhancing [4, 5, 7, 8]. In contrast, control, LMW-HA, or LPS-induced EV contained negligible HA (Figure 4(a)). Considering that the HA receptor, CD44, has previously been reported to be expressed on exosomes [43, 44], we next analyzed HA binding protein expressed in our purified HA-induced EV.  Figure 4(b) indicates that HMW-HA-induced enlargeosomes express the EC barrier enhancing CD44 isoform, CD44s (standard form) [4, 7]. In contrast, LMW-HA-induced exosomes express the EC barrier disrupting HA binding proteins, CD44 isoform CD44v10, and the extracellular serine protease, HABP2 [7, 25]. Basally secreted EVs (control) have low expression of these molecules. In addition, EVs are believed to serve as carriers of RNAs [27, 28].  Figure 4(c) indicates that, compared to control EV, LMW-HA-induced EVs have less total RNA and microRNA while HMW-HA-induced enlargeosomes have ~2-fold higher levels of total RNA and microRNA.

**Figure 5:**
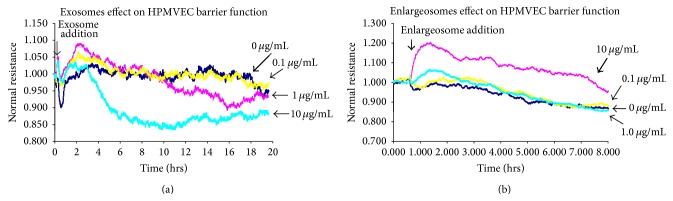
The effects of isolated LMW-HA and HMW-HA-induced EV on human EC barrier function. Panel (a): HPMVEC were plated on transendothelial electrical resistance (TER) electrodes, grown to confluence, and switched to serum-free media and 0, 0.1, 1, or 10 *μ*g/mL isolated LMW-HA-induced exosomes were then added. Addition of isolated LMW-HA-induced exosomes to human EC monolayers promoted barrier disruption in a dose-dependent manner. Panel (b): HPMVEC were plated on transendothelial electrical resistance (TER) electrodes, grown to confluence, and switched to serum-free media and 0, 0.1, 1, or 10 *μ*g/mL isolated HMW-HA-induced enlargeosomes were then added. Addition of isolated HMW-HA-induced enlargeosomes to human EC monolayers induces barrier enhancement in a dose-dependent manner.

Since we observed differential expression of bioactive agents in EV in Figure 4 that could impact vascular integrity, we next determined the role(s) of isolated HA-induced EV in human EC barrier function. HPMVEC were plated on transendothelial electrical resistance (TER) electrodes, grown to confluence, and switched to serum-free media and either isolated LMW-HA-induced exosomes or isolated HMW-HA-induced enlargeosomes were then added.  Figure 5(a) indicates that addition of isolated LMW-HA-induced exosomes to human EC monolayers promotes barrier disruption in a dose-dependent manner. In contrast, Figure 5(b) indicates that addition of isolated HMW-HA-induced enlargeosomes to human EC monolayers induces barrier enhancement in a dose-dependent manner.

**Figure 6:**
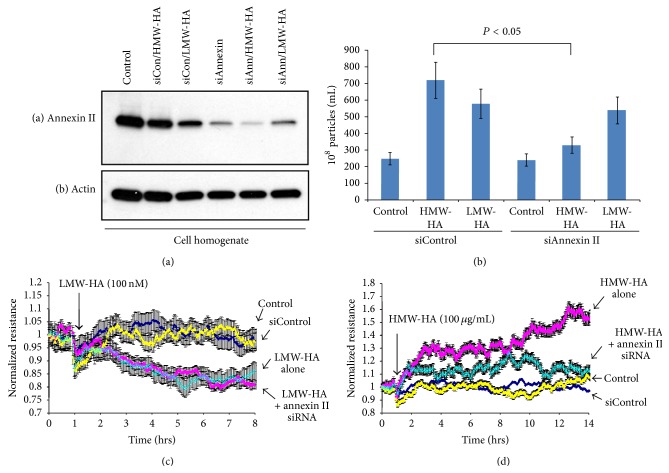
Inhibiting enlargeosome release attenuates HMW-HA-mediated sustained human EC barrier enhancement. Panel (a): inhibition of annexin II expression using siRNA in HPMVEC. Human EC were plated at ~50% confluence and treated with either no siRNA (control), siControl, or siAnnexin II with or without 100 nM LMW-HA or 100 nM HMW-HA for 48 hours. EC lysates were then obtained, run on SDS-PAGE, and immunoblotted with anti-annexin II (a) or anti-actin (b) antibodies. Panel (b): graphical representation of the role of annexin II inhibition in HA-mediated EV production from human EC. HPMVEC were treated as described in Panel (a) and EVs were analyzed utilizing nanosight nanoparticle tracking analysis (NTA). Silencing of annexin II, which has previously been reported to be crucial for enlargeosome exocytosis [23], significantly reduces HMW-HA-, but not LMW-HA-, mediated EV secretion from human EC with *n* = 3 per group and error bars = standard deviation. Panel (c): HPMVEC previously treated with either no siRNA (control), siControl, or annexin II siRNA were plated on transendothelial electrical resistance (TER) electrodes, grown to confluence, and switched to serum-free media and 100 nM LMW-HA was then added. Silencing of annexin II did not affect the sustained human EC barrier disruptive effects of LMW-HA with *n* = 3 per group and error bars = standard deviation. Panel (d): HPMVEC previously treated with either no siRNA (control), siControl, or annexin II siRNA were plated on transendothelial electrical resistance (TER) electrodes, grown to confluence, and switched to serum-free media and 100 nM HMW-HA was then added. In contrast to LMW-HA, silencing annexin II almost completely inhibited the sustained barrier enhancing effects of HMW-HA with *n* = 3 per group and error bars = standard deviation.

To determine whether HMW-HA-induced enlargeosomes affect sustained human EC barrier function, we silenced the expression of annexin II which has previously been reported to be crucial for enlargeosome exocytosis [23].  Figure 6(a) indicates that we can successfully inhibit annexin II expression with siRNA in human EC. Silencing of annexin II significantly reduces HMW-HA-, but not LMW-HA-, mediated EV secretion from human EC (Figure 6(b)). Importantly, silencing of annexin II did not affect the sustained human EC barrier disruptive effects of LMW-HA (Figure 6(c)) but almost completely inhibited the sustained HMW-HA barrier enhancing effects (Figure 6(d)).

## 4. Discussion

Since defects in vascular integrity are an initiating factor in several disease processes, understanding the mechanism(s) of endothelial barrier function can have important clinical implications. We have previously determined that the glycosaminoglycan, hyaluronan (HA), initiates rapid endothelial signal transduction events leading to changes in barrier function. In this study, we investigated the potential long-term mechanism(s) of HA-mediated endothelial cell (EC) barrier regulation. We have identified that ~6 hours after HA addition, human EC differentially release extracellular vesicles (EVs) that contain caveolin-1 and are regulated by caveolin-enriched microdomains (CEM). Utilizing several novel techniques including atomic force microscopy and nanosight nanoparticle tracking analysis (NTA), we have characterized these EVs as a novel type of EV called enlargeosomes (for HMW-HA) and exosomes (for LMW-HA, produced in disease states from HMW-HA by hyaluronidases and ROS). Importantly, isolated LMW-HA-induced exosomes promote EC barrier disruption while isolated HMW-HA-induced enlargeosomes induce EC barrier enhancement. Inhibiting HMW-HA-induced enlargeosome release reduces the sustained barrier enhancing properties of HMW-HA. These findings demonstrate an important mechanism of HA-induced sustained vascular integrity through differential release of EV from human EC.

We have previously reported that CEM are important plasma membrane microdomains involved in rapid protein recruitment and signal transduction leading to endothelial barrier regulation [4–8, 41, 45]. The results of the current study expand the role of CEM to include regulation of HA-induced EV release from human EC. Specifically, our data indicate that inhibiting CEM attenuates both LMW-HA-induced exosome release and HMW-HA-induced enlargeosome release. Further, these EVs contain caveolin-1, a crucial component of CEM [46, 47]. Interestingly, although basally secreted control EVs also contain caveolin-1, CEM do not appear to be involved in their secretion. The fact that these control EVs do not contain markers for exosomes or enlargeosomes indicates that they belong to a different class of EV, possibly exosome-like vesicles or apoptotic bodies [28]. Further analysis is needed to determine the identity of these EVs.

HMW-HA treatment of human pulmonary microvascular EC (HPAEC) causes recruitment of the Ca^2+^-dependent phospholipid and actin-binding protein, annexin II, and the scaffolding protein, AHNAK, to CEM [5]. These proteins are biomarkers of enlargeosomes which are specialized vesicles that occur intracellularly, fusing to the plasma membrane and shedding from the plasma membrane [21–23]. They are implicated in membrane repair which is consistent with their role in sustained HMW-HA-mediated EC barrier enhancement. It is interesting to note the difference in surface topology of control EV and exosomes compared to enlargeosomes. Control EV and exosomes are round in shape and have a relatively smooth surface. In contrast, enlargeosomes are relatively round in shape but have a rough uneven surface topology potentially indicating greater surface protein expression. Control EV and exosomes have a diameter of ~50 nm which is consistent with the accepted exosome size range of 30–100 nm [20, 28]. HMW-HA-induced enlargeosomes have increased size in comparison to exosomes but do not reach the range of larger EV including microsomes [28]. Further, it has been reported that, in astrocyte stellation, there is a Rac1-dependent remodeling of the cytoskeleton with concomitant exocytosis of enlargeosomes [48]. Considering our previous published data that HMW-HA induces Rac1 activation in EC [7], it is possible that Rac1 might also play a role in HMW-HA mediated enlargeosome exocytosis to enhance EC barrier function. In another cellular function, neurite outgrowth, enlargeosomes, and another distinct type of exocytotic vesicle are required for the enlargement of the cell surface [49, 50]. Therefore, we cannot rule out the cooperation of distinct EVs during EC barrier regulation.

Another important result of the current study is that EVs differentially express HA and HA binding proteins which can potentially contribute to their differential EC barrier regulatory effects. Control EV and EV from barrier disruptive agents (LMW-HA and LPS) did not appear to have significant amounts of HA. In contrast, the novel HMW-HA-induced enlargeosomes, in which little compositional information is known, contain HA of ~1 million Da which can potentially contribute to the paracellular propagation of the sustained HMW-HA endothelial barrier enhancing response [5]. Our data indicate that there is differential expression of CD44 isoforms in EV induced by LMW-HA versus HMW-HA. Alternative splicing of the CD44 transcript can give rise to a number of CD44 variants, which express an extra insert in their extracellular membrane proximal domain [51]. HMW-HA-induced enlargeosomes mainly express the barrier enhancing, CD44s (standard form, which lacks extra exon insertions), while LMW-HA-induced exosomes express CD44v10 (variant 10) which contains an extra exon insert and is involved in EC barrier disruption [4]. The precise role of differential CD44 isoform expression in EV function is currently being investigated in our laboratory. In addition, LMW-HA-induced exosomes express the extracellular serine protease, hyaluronan binding protein 2 (HABP2) which we have previously demonstrated to promote endothelial barrier disruption through activation of protease-activated receptor- (PAR receptor-) mediated signal transduction [25]. We are currently examining the expression of HABP2 and another class of HA binding proteins, hyaluronidases, in HA-induced endothelial EV function [25, 52].

MicroRNAs are small noncoding RNA molecules (~22 nucleotides) that function in RNA silencing and posttranscriptional regulation of gene expression [53–55]. Our data indicate that HA-induced EVs contain RNA with a significant fraction of microRNAs. Specifically, compared to control EV, LMW-HA-induced EVs have less total RNA and microRNA while HMW-HA-induced enlargeosomes have ~2-fold higher levels of total RNA and a higher percentage of microRNAs. Considering the pleotropic effects of these molecules, it is plausible to speculate that differential expression of microRNAs in LMW-HA-induced exosomes and HMW-HA-induced enlargeosomes can contribute to differential endothelial barrier function. Indeed, it has been reported that EC treated with hypoxia, TNF-alpha, high glucose, and mannose produce exosomes with altered protein and RNA content [56]. Further, curcumin treatment of EC produces exosomes that promote EC barrier function [57] while cancer-secreted exosomes containing miR-105 inhibit EC barrier function and promote metastasis [58].

In conclusion, our findings demonstrate an important mechanism of HA-induced sustained vascular integrity through CEM-regulated differential release of EV from human EC. Specifically, LMW-HA treatment of HPMVEC induces release of endothelial barrier disruptive exosomes while HMW-HA promotes secretion of a novel type of EV, enlargeosomes, which promote sustained endothelial barrier enhancement. Further, these EVs contain differential expression of HA, HA binding proteins, and miRNAs which can contribute to their barrier regulatory properties. Utilization of isolated HMW-HA-induced specialized enlargeosomes and/or their bioactive components can be a potential therapeutic strategy for diseases involving impaired vascular integrity.
